# Virtual memory cells make a major contribution to the response of aged influenza-naïve mice to influenza virus infection

**DOI:** 10.1186/s12979-018-0122-y

**Published:** 2018-08-08

**Authors:** Kathleen G. Lanzer, Tres Cookenham, William W. Reiley, Marcia A. Blackman

**Affiliations:** Trudeau Institute, 154 Algonquin Avenue, Saranac Lake, NY 12983 USA

**Keywords:** T cell receptor repertoire, Virtual memory (VM) T cells, True memory (TM) T cells, Influenza, Ageing, Mouse model

## Abstract

**Background:**

A diverse repertoire of naïve T cells is thought to be essential for a robust response to new infections. However, a key aspect of aging of the T cell compartment is a decline in numbers and diversity of peripheral naïve T cells. We have hypothesized that the age-related decline in naïve T cells forces the immune system to respond to new infections using cross-reactive memory T cells generated to previous infections that dominate the aged peripheral T cell repertoire.

**Results:**

Here we confirm that the CD8 T cell response of aged, influenza-naïve mice to primary infection with influenza virus is dominated by T cells that derive from the memory T cell pool. These cells exhibit the phenotypic characteristics of virtual memory cells rather than true memory cells. Furthermore, we find that the repertoire of responding CD8 T cells is constrained compared with that of young mice, and differs significantly between individual aged mice. After infection, these virtual memory CD8 T cells effectively develop into granzyme-producing effector cells, and clear virus with kinetics comparable to naïve CD8 T cells from young mice.

**Conclusions:**

The response of aged, influenza-naive mice to a new influenza infection is mediated largely by memory CD8 T cells. However, unexpectedly, they have the phenotype of VM cells. In response to de novo influenza virus infection, the VM cells develop into granzyme-producing effector cells and clear virus with comparable kinetics to young CD8 T cells.

**Electronic supplementary material:**

The online version of this article (10.1186/s12979-018-0122-y) contains supplementary material, which is available to authorized users.

## Background

A diverse repertoire of naïve T cells is thought to be necessary for an optimal response to infections [1–6]. With age, the numbers of naïve T cells decline, such that the ratio of memory-phenotype to naïve T cells in the periphery greatly increases. In addition, the repertoire diversity becomes constrained [7–15]. The decline of the naïve repertoire of CD8 T cells with age is a consequence of reduced thymic output, increasing antigen experience, peripheral homeostatic proliferation and the development of large clonal expansions of cells displaying a memory phenotype [16–21].

The decline in naïve T cells with aging has been correlated with impaired immunity and reduced ability to respond to new infections [3–6, 13, 22, 23]. Consistent with this, our previous studies confirmed that declining numbers of naïve CD8 T cells in aged mice correlated with poor responses to de novo infection with influenza virus [7]. Specifically, the response to an immunodominant nucleoprotein epitope (NP_366_), but not the co-dominant epitope (PA_224_), was found to be dramatically reduced in aged mice. We further showed that the naïve precursor frequency of NP-specific CD8 T cells was 10-fold lower than PA-specific CD8 T cells in aged mice, providing an explanation for the selective decline in the immune response to influenza virus NP. This study provided proof of concept that the naïve repertoire to epitopes with a low precursor frequency may become so constrained during aging that “holes” develop in the repertoire [7].

With increasing antigen experience during the lifespan and the decline in numbers and diversity of naïve T cells, we have hypothesized that memory CD8 T cells generated in response to previous antigen exposure and that are fortuitously cross reactive make a major contribution to T cell responses to de novo infections in aged mice [6]. Consistent with this hypothesis, unexpected cross-reactivity has been demonstrated between CD8 T cells specific for distinct epitopes expressed by different viruses [24–31]. It has also been shown that CD4 T cells respond to antigens to which the individual has never been exposed, as a consequence of cross-reactivity [32]. Together, the data show that T cell recognition of antigen/MHC is highly degenerate, and T cell responses exhibit extensive and unexpected cross reactivity [5, 33].

Fortuitously cross-reactive memory CD8 T cells provide a potential explanation of how protection can be maintained within aged mice as the naïve repertoire declines. One prediction of this hypothesis is that the CD8 T cell response to new infections in aged mice would be likely to exhibit reduced repertoire diversity compared to CD8 T cell responses in young mice. In addition, the specific and perhaps unique prior antigenic experience and repertoire of memory cells in each individual would result in heterogeneous responses in individual aged animals. Another prediction of the hypothesis is that the reduced repertoire diversity of the fortuitously cross reactive memory T cell responses would result in impaired immunity and delayed viral clearance in aged mice [6]. The goal of the current study was to test these possibilities.

Conventional memory CD8 T cells can be classified into three distinct types that are distinguished by phenotypic markers and trafficking patterns. One population, effector memory cells (EM), express low levels of CD62L and CCR7, lack the ability to home to lymph nodes, and preferentially localize to peripheral tissues. A second population, central memory cells (CM), express high levels of CD62L and CCR7, and circulate through the blood and secondary lymphoid organs. A third population, resident memory cells (T_RM_), express CD69 and CD103, reside in the peripheral tissues, and do not circulate [34–37].

Memory cells were originally defined as a population of long-lasting cells generated by exposure to antigen. However, recently it has become apparent that antigen-specific, memory-phenotype T cells can also develop in the absence of antigenic stimulation. For example, antigen-specific memory phenotype T cells can be detected by antigen/MHC tetramers from naïve and germ-free mice. In one study, CD8 T cells specific for three different peptides from ovalbumin, vaccinia virus and herpes simplex virus were isolated from naïve mice and, unexpectedly, a significant percentage of the cells were of a memory phenotype (CD44^High^) [38]. There are two major subsets of these antigen-inexperienced memory cells, termed innate memory and virtual memory (VM) [39]. These subsets are difficult to distinguish phenotypically, as both populations of antigen-inexperienced memory cells are characterized by their high expression of CD62L and CD122, and low expression of CD49d. However, there are developmental and functional differences between the two subsets of cells [39]. Importantly for this study, there are relatively few innate memory cells in C57BL/6 mice [39, 40], and the frequency of VM cells have been shown to increase with age, such that the majority of CD8 T cells in aged C57BL/6 mice, used in this study, have a VM phenotype [2, 41, 42]. These data raise the possibility that VM cells contribute to the response of aged, influenza-naïve mice to de novo influenza infection.

In this study we have tested the hypothesis that cross-reactive memory T cells not specifically elicited by previous infection play a major role in the response of aged mice to new infections. As predicted, we show that the response of aged, influenza-naive mice to a new influenza infection is mediated largely by memory CD8 T cells that exhibit reduced repertoire diversity that is heterogeneous in individual mice. However, unexpectedly, they have the phenotype of VM cells. In response to de novo influenza virus infection, the VM cells develop into granzyme-producing effector cells and clear virus with comparable kinetics to young CD8 T cells.

## Results

### Memory CD8 T cells from aged, influenza-naïve mice respond to de novo infection with influenza virus and produce only minimal responses to influenza NP

**Fig. 1:**
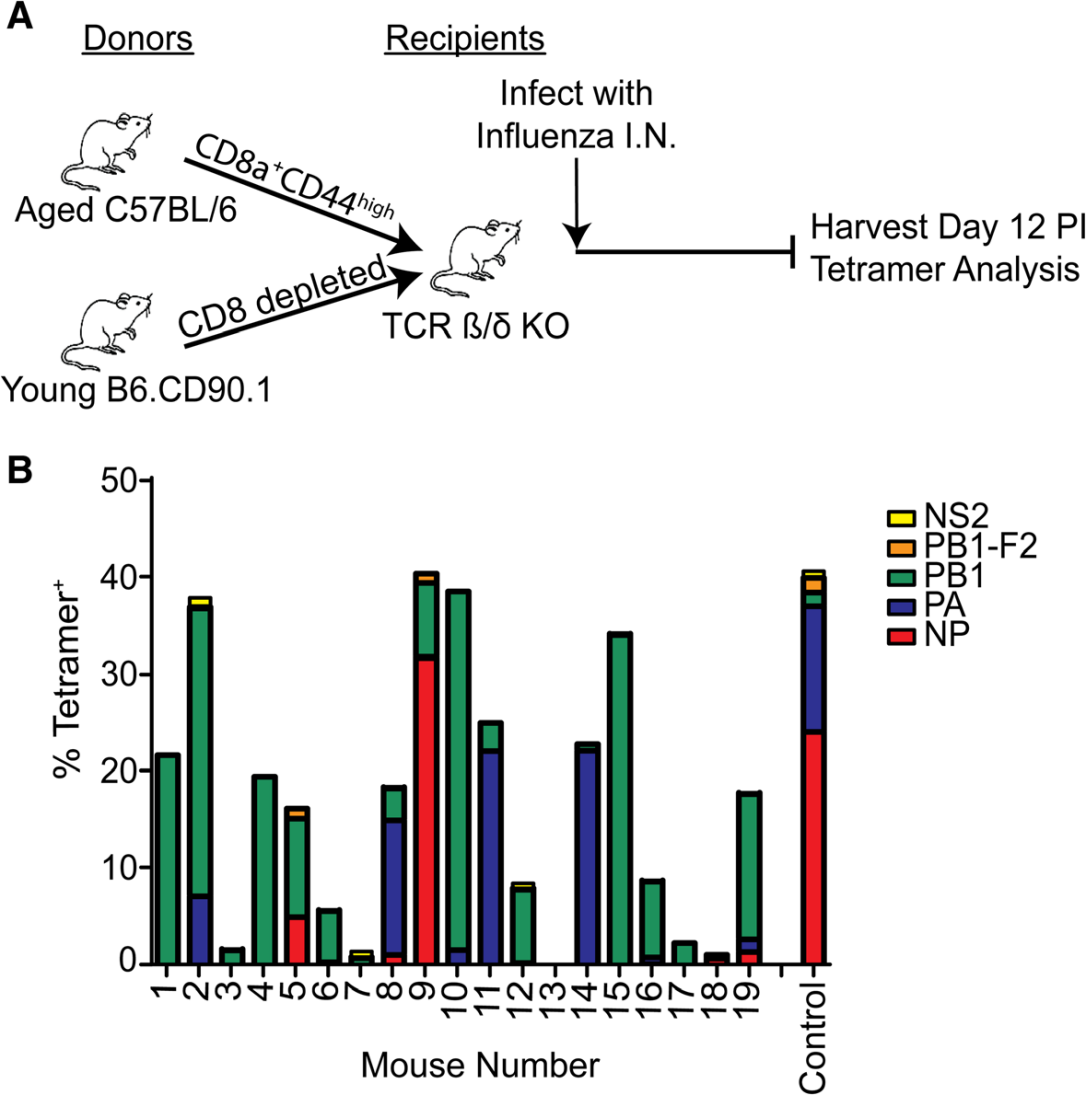
Memory cells from naïve aged mice can respond to de novo infection with influenza. **a** Sorted CD44^High^ memory CD8 T cells from naïve aged (18–22 months) mice were co-transferred with CD8 T cell-depleted splenocytes from young mice into T cell deficient TCR βδ−/− mice, infected with influenza virus, and the responding CD8 T cells analyzed at 12 days post infection. **b** The epitope-specific CD8 T cell response of 19 individual aged mice is indicated by colors and a young WT control mouse is shown

To demonstrate that memory CD8 T cells from aged mice that had not previously been exposed to influenza virus were capable of responding to influenza virus, we FACS-sorted memory CD8 T cells (CD44^High^) from aged, naïve specific pathogen free mice and adoptively transferred them into young T cell-deficient (TCR βδ −/−) mice. The following day the mice were infected with influenza virus and on day 12 post infection (p.i.) the responding CD8 T cells were tested for reactivity to a panel of five MHC class I influenza-specific epitopes from the viral proteins - nucleoprotein (NP_366_/D^b^), acid polymerase (PA_224_/D^b^), basic polymerase I (PB1_703_/K^b^), the F2 of PB1 (PB1 F2_62_ /D^b^) and non-structural protein 2 (NS2_114_/K^b^) Fig. 1a. It has previously been shown in young mice that the antigen-specific T cell response to the NP_366_/D^b^ and the PA_224_/D^b^ epitopes are relatively equi-dominant following primary infection with influenza virus (see a representative control response in Fig. 1b), whereas the secondary antigen-specific T cell response is sharply biased toward the NP_366_/D^b^ epitope [43–45]. In contrast, we have demonstrated a dramatically reduced response to NP_366_/D^b^ in aged mice [7]. Consistent with this, analysis of adoptive transfers from 19 individual aged mice showed that the pattern of response to the two epitopes was heterogeneous in individual mice and was not consistent with what was typically seen in either primary or recall responses in young mice (Fig. 1b). In only one of the 19 adoptive transfers of aged mouse cells did we observe a dominant NP_366_/D^b^ response after influenza infection. In three additional mice we observed a PA_224_/D^b^ dominant response, and in nine mice we observed a PB1_703_/K^b^-dominant response (which in a young intact SPF mouse represents a sub-dominant epitope). These adoptive transfer data are consistent with our previous data showing a selective reduction in the ability of aged mice to mediate a response to the immunodominant NP_366_/D^b^ epitope after influenza infection, which is highly variable in individual mice [7]. In order to ensure that the development of the immunodominant NP_366_/D^b^ response in the aged mice was not impaired due to lack of some deficiency in the TCR βδ −/− mice, such as CD4 T cell help, we co-transferred CD8-depleted splenocytes from young, congenically disparate mice along with the aged memory CD8 T cells. The number of sorted CD44^High^ memory cells transferred from individual mice was variable, depending upon the yield of cells from the sort, but this did not correlate with the reduction of the NP_366_/D^b^ response (Additional file 1).

While these findings demonstrate skewing of the epitope diversity within the aged memory populations of cells, it is important to also take into consideration that T cell clonal expansions increase with aging and can be a contributing factor to the declining ratio of naïve:memory CD8 T cells. These clonal expansions could be a significant component of the transferred CD44^High^ cells [20, 21, 46, 47]. Therefore, to eliminate this complication in the current experiments, we routinely pre-screened aged mice and eliminated those mice with TCE, determined as described in the Materials and Methods.

### Competition between memory and naïve CD8 T cells from aged mice responding to de novo influenza virus infection

**Fig. 2:**
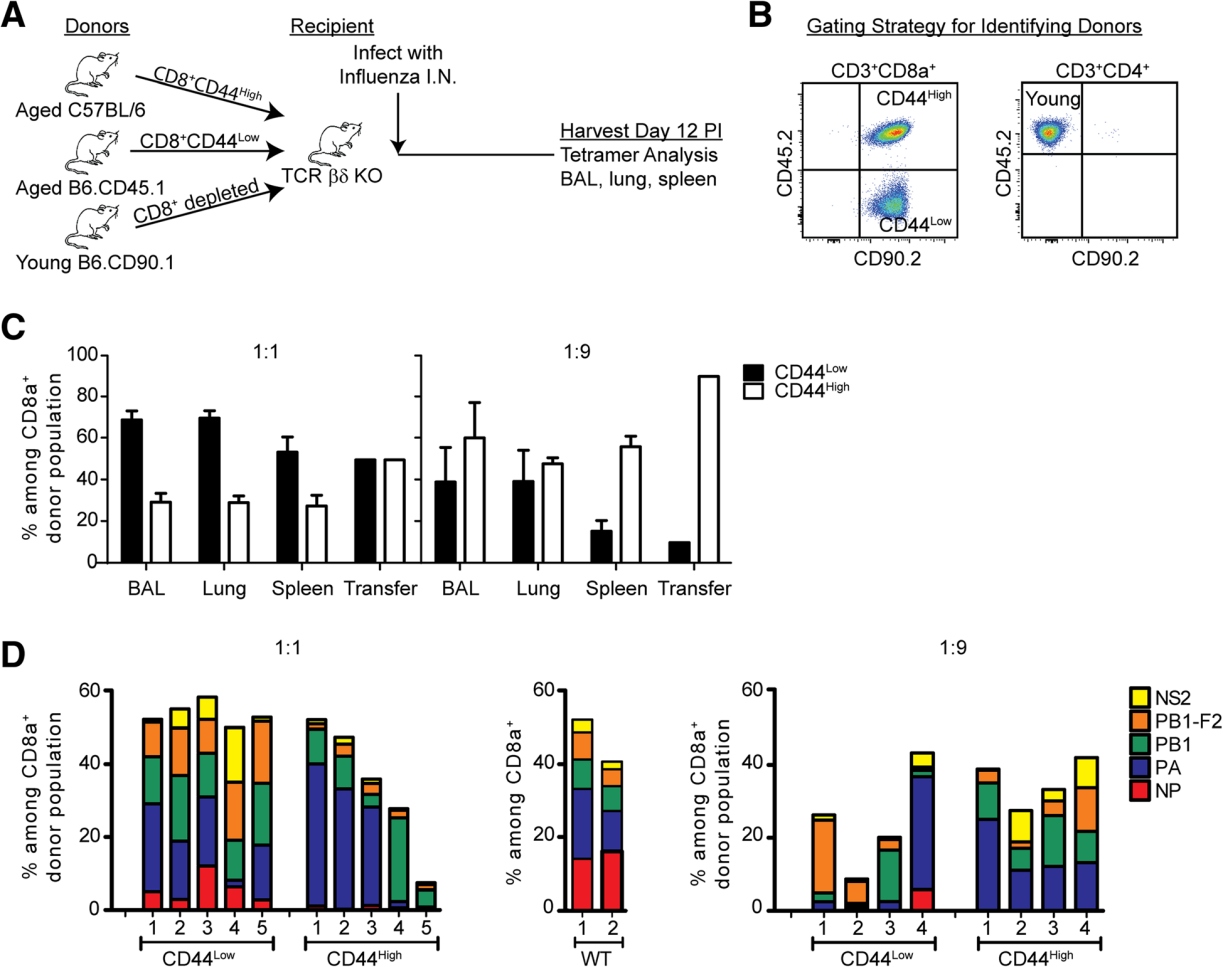
Contribution of memory phenotype cells from naïve aged mice to new infections. **a** Sorted CD44^Low^ CD8 T cells from naïve aged mice (18–22 months) were co-transferred with sorted CD44^High^ CD8 T cells from naive aged mice in a 1:1 or a 1:9 ratio, with CD8 T-cell depleted splenocytes from young mice (2–3 months), into T cell deficient TCR βδ−/− mice. The mice were infected with influenza virus, and the responding CD8 T cells originating from naïve or memory donors analyzed at 12 days post infection. **b** Flow cytometry gating strategy used to identify the donor populations in the lung: distinguished by CD90.2^+^CD45.2^+^ cells for donor aged C57BL/6, CD90.2^+^CD45.2^−^ for donor aged B6.CD45.1, and CD90.2^−^CD45.2^+^ for donor young B6.CD90.1. **c** The response of donor naïve and memory CD8 T cells is shown for the 1:1 transfer (left) and the 1:9 transfer (right). **d** The epitope-specificity of the donor naive and memory CD8 T cell response, indicated by colors, is shown for the 1:1 transfer (left), the 1:9 transfer (right), and the memory response from intact wild type (WT) mice (middle). The data represent two independent experiments

The previous data show that memory CD8 T cells from influenza-naïve, aged mice could respond to influenza virus infection when they were transferred in isolation and were the only source of CD8 T cells. However, in order to understand whether these cells could dominate the response in the context of the total population of peripheral CD8 T cells from aged mice, it was important to determine the ability of these cells to compete with naïve CD8 T cells. Therefore, we co-transferred naïve (CD44^Low^) and memory (CD44^High^) cells from aged mice into TCR βδ −/− mice (as previously) in a 1:1 ratio (Fig. 2a). Cells were isolated from congenically distinct mice (Fig. 2b), to allow for the identification of naïve and memory donor cells during the response. In an additional set of experiments, we co-transferred naïve and memory CD8 T cells in a 1:9 ratio, more typical of the ratio in aged mice. In both cases, as previously, we co-transferred CD8-depleted spleen cells from young mice, distinguished by a third congenic marker (see Figure legend for details). The data (Fig. 2c) show that memory CD8 T cells made a minor contribution to de novo influenza infection in the bronchoalveolar lavage (BAL), lung tissue and spleen when the naïve and memory cells were transferred in a 1:1 ratio, and a greater contribution to the response when the cells were transferred in a 1:9 ratio, a ratio more reflective of their natural distribution in aged mice. These data show that cross-reactive memory cells in aged, influenza-naive mice can make a major contribution to the response to a de novo influenza virus infection.

We also analyzed the epitope specificity of the response within these competitive transfer experiments (Fig. 2d). The repertoire of naïve CD8 T cells in the 1:1 transfer was diverse, and consisted of responses to all 5 epitopes tested, reflective of the repertoire of wild type mice. In contrast, the repertoire of the responding memory cells in both the 1:1 and the 1:9 transfer was skewed from the normal response of naïve CD8 T cells, and varied greatly in individual mice. Together, these data support the hypothesis that cross-reactive memory CD8 T cells, not generated in response to influenza virus infection, make a major contribution to the response to a newly-encountered antigen in aged mice, with skewed repertoire diversity.

### Distribution of peripheral memory CD8 T cells in influenza memory mice infected when young or aged

**Fig. 3:**
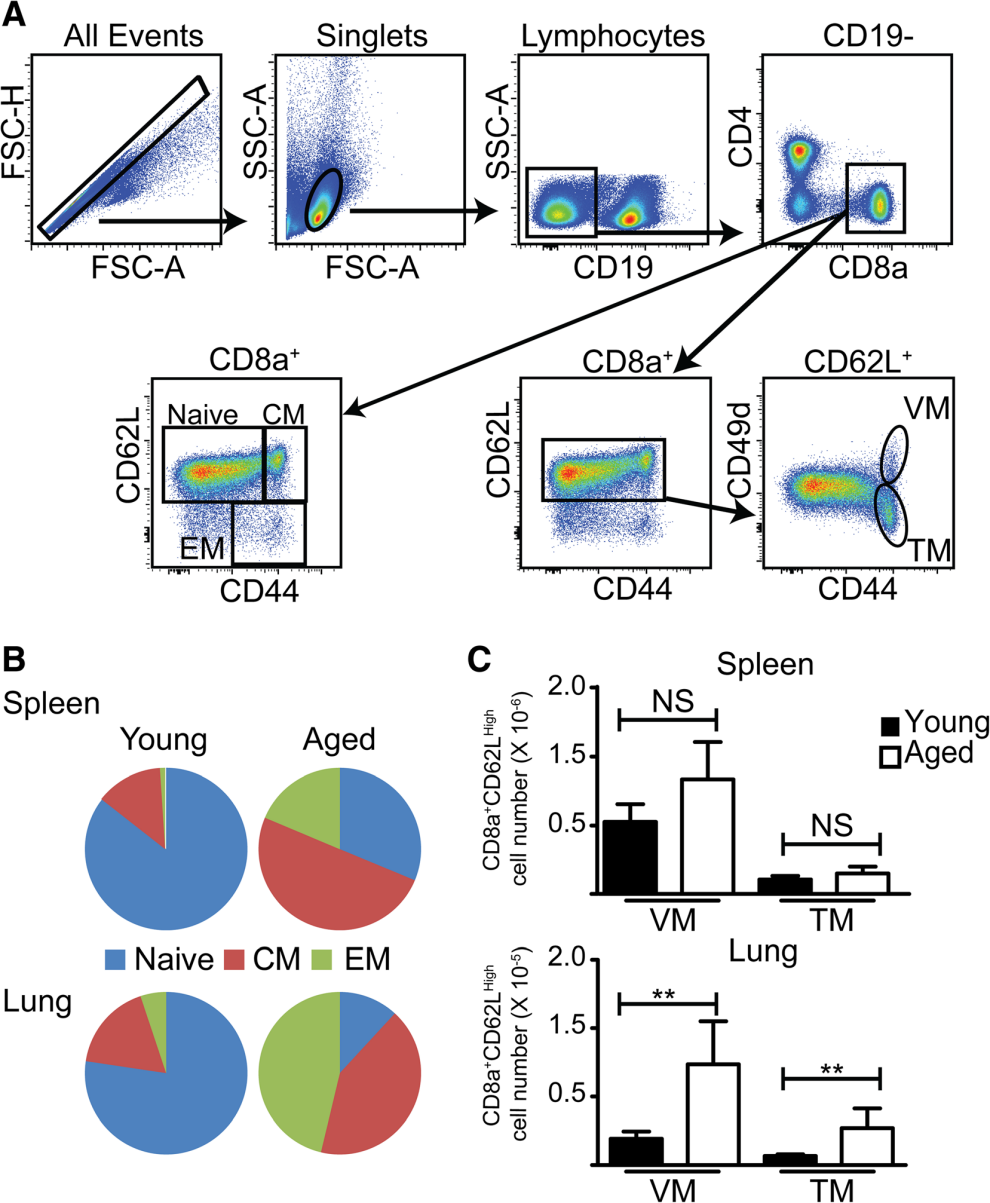
Distribution of peripheral CD8 T cells in naive young and aged mice. **a** Representative flow cytometry staining from spleen illustrating the gating strategy for identifying CD8 subsets in B and C. **b** The distribution of naïve (CD62L^High^/CD44L^Low^), CM (CD62L^High^/CD44^High^) and EM (CD62L^Low^/CD44^High^) in the spleen and lung of young (2–3 months) and aged (18–22 months) SPF mice. **c** The number of VM (CD49d^Low^) and TM (CD49d^High^) among the CM population in the spleen and lung of young and aged mice. Bars represent the mean ± SD. The data are from a single experiment, *n* = 5 mice/group, and are consistent with published data [42]. Data were analyzed by unpaired, two-tailed T test (**p < 0.01)

The previous data supported our hypothesis that in aged mice the naïve CD8 T cell repertoire may become so constrained that responses to newly-encountered pathogens are mediated primarily by fortuitously cross reactive memory CD8 T cells, previously generated in response to unrelated antigens. The composition of the peripheral CD8 T cells in the spleen and lung of young mice is predominately of a naïve phenotype (CD44^Low^), whereas the composition in aged mice is predominantly a memory phenotype (CD44^High^), consistent with published data [42] (Fig. 3). Furthermore, memory phenotype cells in young mice were predominantly CM whereas aged mice had a large percentage of EM in addition to CM. Further analysis of the phenotype of the CM using the phenotypic markers CD62L and CD49d showed that cells with a virtual memory (VM) phenotype dominated the CM population in both young and aged mice. We termed the memory cells without a VM phenotype (CD49d^High^), true memory (TM) cells. The low number of TM in aged mice raised the possibility that memory T cells of a VM rather than a TM phenotype responded to de novo influenza infection. Thus we next investigated whether the adoptively-transferred CD8 memory T cells responding to a primary influenza infection in aged mice originated from VM or TM cells.

### VM dominate the response of memory CD8 T cells from aged naïve mice to primary influenza virus infection

**Fig. 4:**
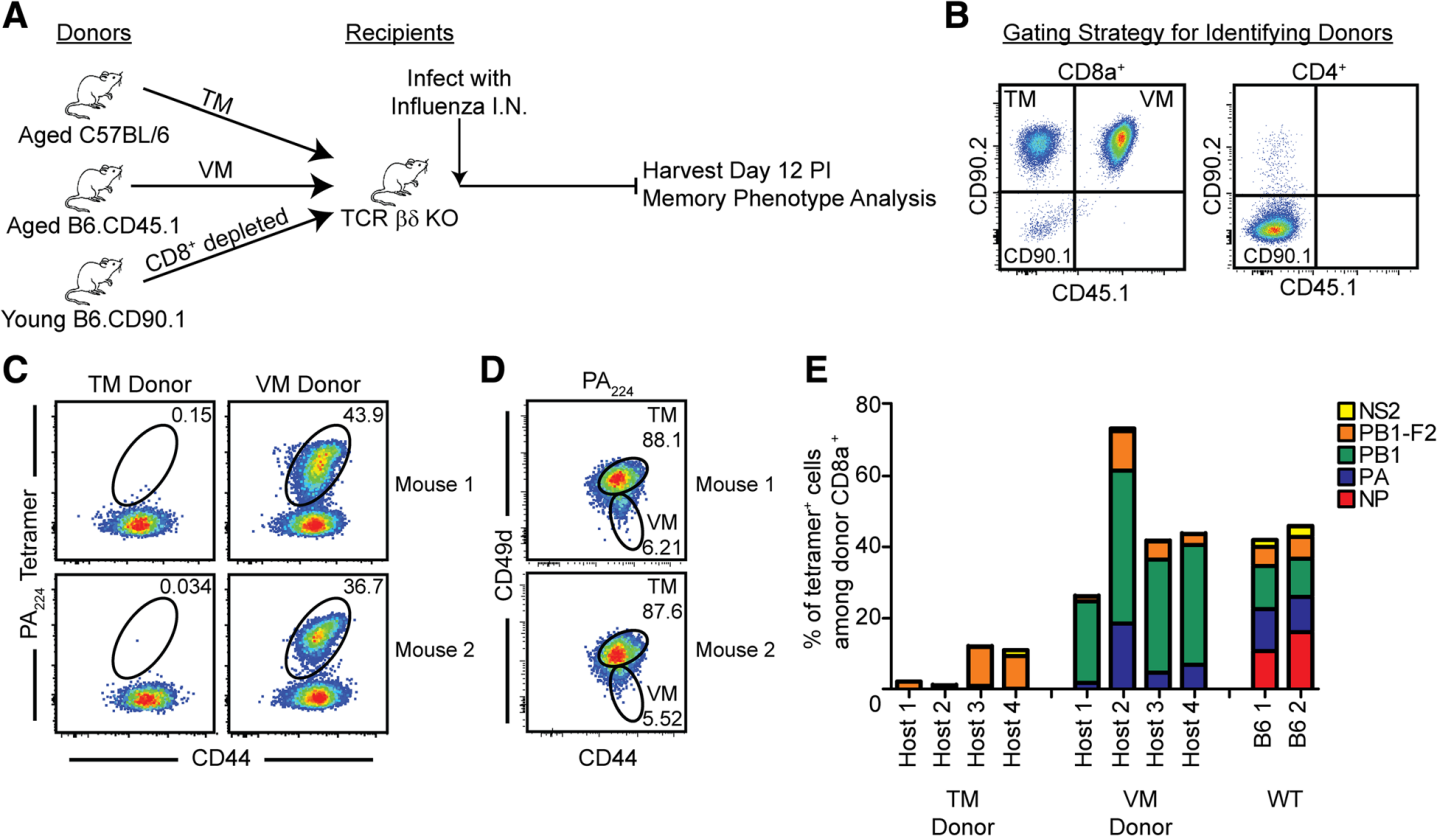
VM cells dominate the memory response of naïve aged mice to de novo virus infection. **a** CD8 TM (CD44^High^/CD49d^High^) and VM (CD44^High^/CD49d^Low^) cells were FACS-sorted from naïve aged C57BL/6 and naïve aged B6.CD45.1 mice, respectively, and adoptively transferred into T cell deficient TCR βδ−/− mice at a 1:1 ratio, with CD8 depleted splenocytes from naïve young B6CD90.1 mice (2–3 months). The mice were infected with influenza virus and the responding CD8 T cells originating from TM or VM donors analyzed at 12 days post infection. **b** Flow cytometry gating strategy used to identify the donor populations in the lung: distinguished by CD90.2^+^CD45.1^−^ cells for donor aged C57BL/6, CD90.2^+^CD45.1^+^ for donor aged B6.CD45.1, and CD90.2^−^CD45.2^−^ for donor young B6.CD90.1. **c** Representative FACS plots from two individual recipient mice, gated on donor CD8 T cells from the lung. Data show that the response of cells specific for a representative influenza-specific tetramer, PA_224_, was mediated almost exclusively by VM cells. **d** Representative FACS plots from two mice, gated on VM donor PA_224_-specific cells, showed upregulated expression of CD49d. **e** The epitope-specificity of the TM donor, VM donor, and intact WT control memory response are represented by colors. The data represent two independent experiments

To determine if the memory CD8 T cells that were responding during influenza infection of aged mice were of the TM or VM phenotype, the two populations were sorted from aged naïve B6 and congenic (B6.CD45.1) mice based on CD49d expression. Cells were then adoptively transferred into T cell-deficient (TCR βδ^−/−^) mice, along with CD8-depleted T cells from young congenic mice (B6.CD90.1), and infected with influenza virus, as shown (Fig. 4a). We then followed the response to PA_224_/D^b^ since our previous findings demonstrated the low and variable nature of the NP_366_/D^b^ response in aged mice (Figs. 1b and 2d). As shown in Fig. 4c, the influenza antigen-specific T cell response to the PA_224_/D^b^ epitope was dominated by VM cells, with only a very small component arising from the adoptively transferred TM cells. Further examination of the antigen-specific T cell repertoire to the five major influenza epitopes in C57BL/6 mice reinforced the conclusion that the influenza-specific response of aged mice predominantly derives from VM cells (Fig. 4e). We also examined the phenotype of the antigen-specific T cells that developed in response to the influenza infection and found that all of the cells had upregulated CD49d, switching to a TM phenotype (Fig. 4d). Taken together, the data show that the response of memory CD8 T cells from influenza-naïve aged mice to a new influenza infection is predominantly mediated by VM cells and these VM cells then convert to a TM phenotype, consistent with previous observations [48].

### VM cells from aged mice generate functional effector cells

**Fig. 5:**
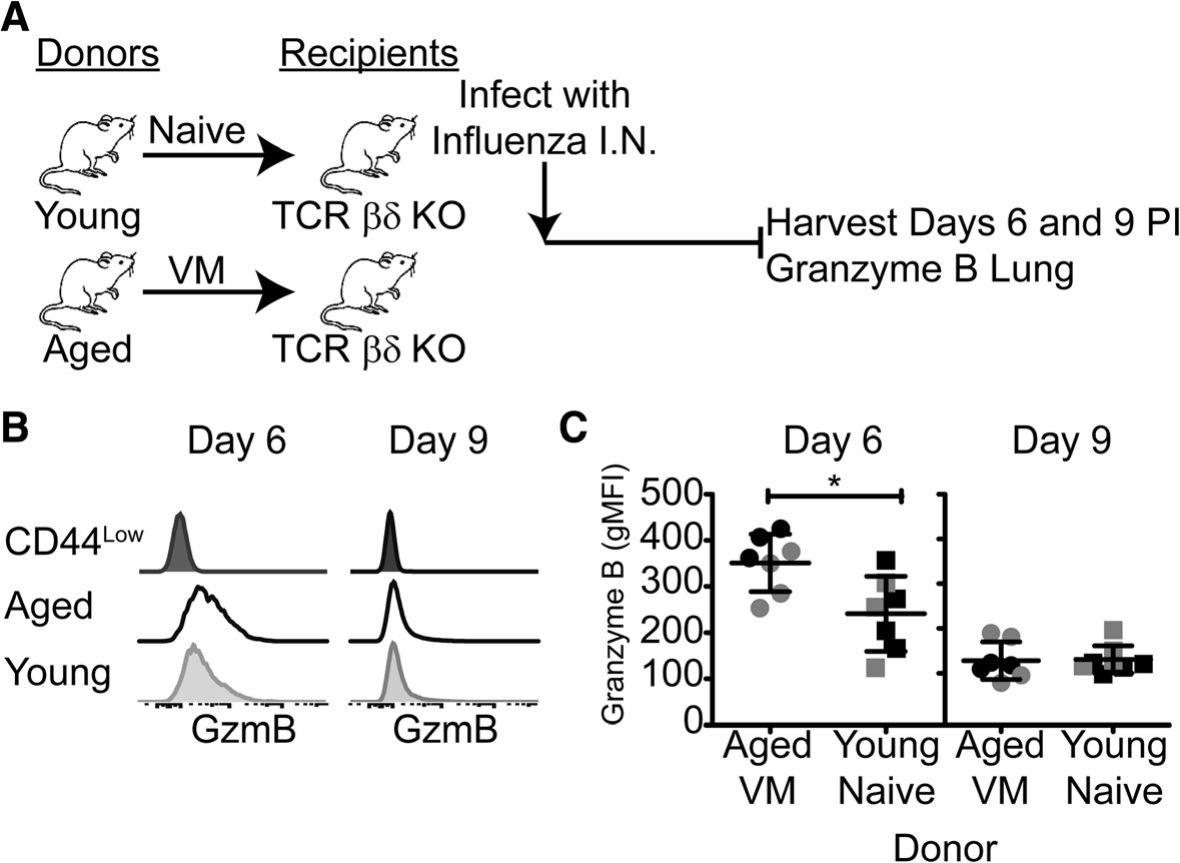
VM cells from aged mice express Granzyme B following response to influenza infection. **a** Pooled CD8 VM cells (CD44^High^/CD62L^High^/CD49d^Low^) from aged C57BL/6 donors and pooled CD8 naïve cells (CD44^Low^/CD62L^High^) from young C57BL/6 donors were FACS-sorted and adoptively transferred into individual T cell deficient TCR βδ−/− mice, infected with influenza, and assessed for Granzyme B expression at days 6 and 9 post infection. **b** Representative histograms gated on the donor CD8 T cell population show Granzyme B expression in the lung at the indicated time points after infection. **c** Summary graphs show plots of of Granzyme B expression (gMFI) in lung donor CD8 T cell populations from individual recipient mice at the indicated times after infection. Data from two independent experiments, pooled for statistical analysis, are represented in separate shades. Bars represent the mean ± SD. Data were analyzed by unpaired, two-tailed T test (*p* < 0.05)

In order to determine whether influenza virus reactive VM CD8 T cells were functional, we analyzed granzyme responses (Fig. 5a). Granzyme staining from representative individual mice is shown in Fig. 5b. The plotted data from two independent experiments (Fig. 5c) show the VM cells from aged, influenza-naïve mice developed a granzyme response at least as strong as that developed by the naïve T cells from young mice at day 6. By day 9 the response of both populations had waned.

**Fig. 6:**
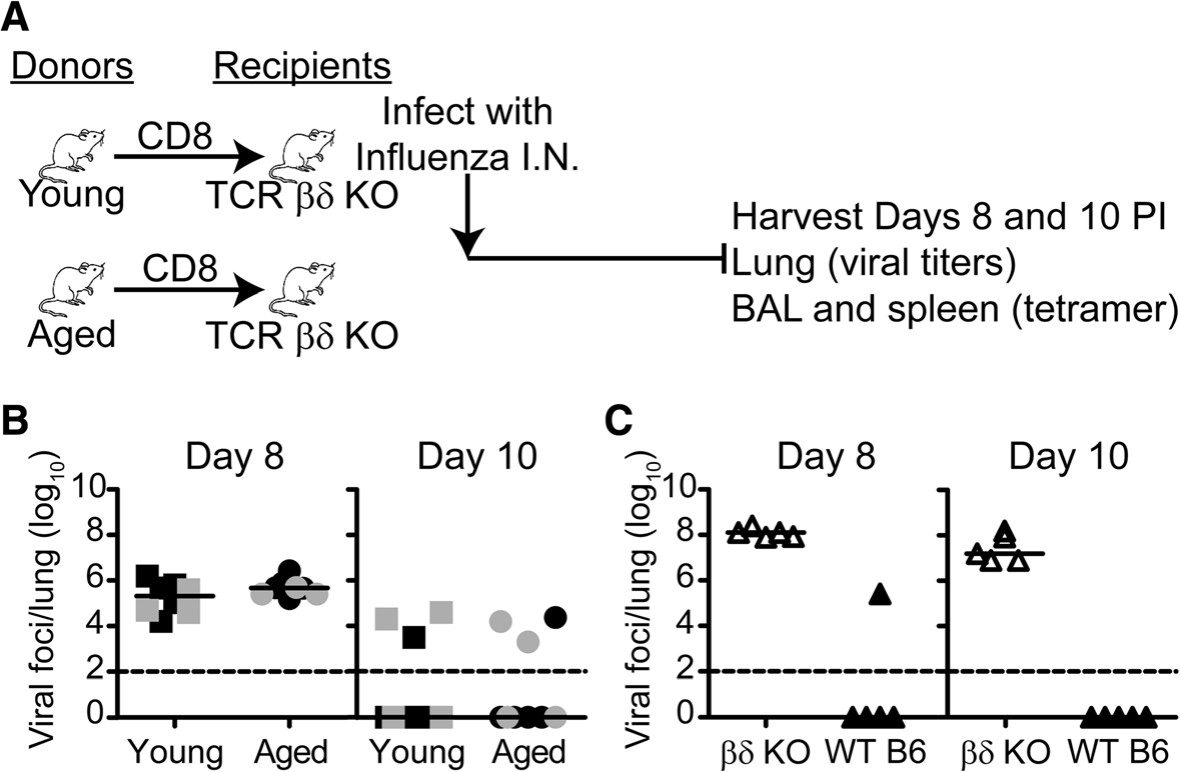
Transferred CD8 cells from naïve aged and young mice clear virus with similar kinetics. **a** CD8 T cells from naive aged or young mice were enriched from splenocytes using negative selection, adoptively transferred into individual T cell deficient βδ−/− mice, infected with influenza virus, and the lungs were harvested at days 8 and 10 post-infection to determine viral titers. **b** Summary graphs of the viral foci/lung on days 8 and 10 post infection. Data from two independent experiments, pooled for statistical analysis, are represented in separate shades. Bars represent the median. Viral titers on day 8 or on day 10 in the two groups did not differ significantly (*p* > 0.05). Data were analyzed by Mann-Whitney Test. **c** Graph of the viral foci/lung on days 8 and 10 post infection for control individual WT C57BL/6 mice and individual T cell deficient βδ−/− mice that did not receive adoptively transferred CD8 T cells

The delay in viral clearance of influenza infection in aged mice has been well established [49]. However, our observations that naïve CD8 T cells from young mice and VM CD8 T cells from aged mice generate comparable granzyme responses prompted us to examine viral clearance (Fig. 6a). Therefore, we compared viral clearance mediated by young CD8 T cells (mostly naïve) and aged CD8 T cells (mostly VM). Unexpectedly, the kinetics of viral clearance in TCR βδ −/− mice into which young or aged CD8 T cells had been transferred was comparable. In order to rule out the possible participation of cytotoxic young CD4 T cells [50, 51], we eliminated young CD4 T cells from the co-transfer. The data (Fig. 6b) showed that the kinetics of viral clearance remained comparable between transferred CD8 T cells from young mice, which have mostly naïve CD8 T cells and aged mice, which have mostly VM CD8 T cells. TCR βδ−/− mice that received no transferred T cells (Fig. 6c) failed to clear virus, indicating that the viral clearance we are detecting in panel B is mediated by the transferred T cells.

Together, our data show that VM effectively produce granzyme and mediate viral clearance. These data are consistent with previous reports that VM protect against infectious challenge and mediate rapid effector function [48, 52, 53], although it is possible that there are kinetic differences compared with TM, which have not been examined (in isolation) in this study.

## Discussion

It is well-established that there is a dramatic reduction in the number of naïve CD8 T cells and a corresponding increase in the number of memory phenotype CD8 T cells in the periphery of aged mice. Because of the decline in numbers (and, consequently, repertoire diversity) of naïve cells, we hypothesized that the response of aged mice to new infections would be dominated by cross-reactive memory cells previously generated in response to other antigens during the lifespan, rather than from the pool of naïve cells [6, 54]. Using an adoptive transfer system, we have shown that, indeed, memory CD8 T cells from aged, influenza-naïve mice are capable of responding to influenza virus and developing into effector T cells that could migrate to the sites of infection. Our dual adoptive transfer experiments showed that when naïve and memory CD8 T cells were co-transferred at a ratio consistent with that found in aged mice (1:9), the memory T cells dominated the response. Yet the ability of adoptively transferred aged CD8 T cells to respond was outcompeted by the naïve CD8 T cells when transferred at the 1:1 ratio. Furthermore, the epitope specificity of the memory T cell response was substantially constricted compared with naïve T cells, and heterogeneous in individual mice, particularly with regard to the normally immunodominant epitope, NP (Fig. 1b). These data are consistent with previous studies by us [7] and others [9], showing reduced repertoire diversity following primary infection of intact aged mice.

Thus, our data support the hypothesis that the response to new antigens in aged mice is dominated by cross-reactive memory CD8 T cells. However, phenotypic analysis revealed that the memory cells were not “true” memory cells (TM), as would have been generated to previous infections. Instead, the memory cells had characteristics of VM, which are generated in the absence of antigen [38, 53, 55, 56]. Importantly, the cells were found to be functional in terms of granzyme production and cleared virus upon influenza infection after adoptive transfer into young, T cell-deficient mice.

VM cells in mice appear in the periphery soon after birth in the absence of antigen [38, 48, 56–58], and accumulate with age in naïve specific pathogen free (spf) mice [2, 41, 42, 59]. While the factors influencing the generation of VM are not completely understood, it has been hypothesized that these cells arise via different mechanisms in young and aged mice. For example, secondary TCRα rearrangements have been shown to be necessary for the age-related dominance of VM, indicating a requirement for TCR ligation in the generation of VM in aged mice, which was not a requirement in young mice [59]. Clearly, the mechanisms involved in VM cell generation, as well as the maintenance of these cells, need to be studied in both young and aged mice.

It has been shown that VM proliferate more robustly than naïve CD8 T cells after antigen stimulation of young mice [38, 48]. However, we observed that aged memory cells were outcompeted by aged naïve CD8 T cells after influenza infection when transferred 1:1. This is consistent with the observation that VM in aged mice exhibit impaired TCR-mediated proliferation [59]. It has also been suggested that the antigen diversity of VM and naïve T cells is similar, because tetramer positive cells were identified in unprimed (young) animals to all epitopes tested [38]. However, this was not observed in our studies, as we saw a greater degree of epitope diversity in the response of naïve compared to VM CD8 T cells following influenza infection of aged mice (Fig. 2d).

Since our results demonstrated that the vast majority of the responding CD8 T cells that develop in the aged mice in response to influenza infection were from the population of VM cells, it was important to determine if these cells were capable of effector function. It has been shown that VM produce IFNγ, both in response to cognate antigen as well as cytokine-mediated, antigen independent stimulation [38, 39, 48]. In addition, VM have been shown to express granzyme, and can mediate antigen-independent bystander killing [39, 53]. Consistent with these published data, our results show that VM cells from aged mice have the capacity to become granzyme-secreting effectors and clear virus with kinetics comparable to CD8 T cells from young, influenza-naïve mice. Interestingly, it has been shown that VM preferentially differentiate into CM after stimulation, whereas TM cells tend to become EM upon secondary challenge [48], suggesting that the response of VM to influenza infection may actually contribute to strong maintenance of memory. This intriguing idea was not directly examined in the current study.

A key question in the field of aging immunity is the relevance of the aged mouse model to elderly humans. Whereas naïve CD8 T cell repertoire diversity in the mouse declines to the extent that the ability to respond to new infections is impaired [3–6, 13, 22, 23] and “holes” in the repertoire can develop [7], it has been shown that there is only a modest (3–5-fold) reduction in CD8 T cell repertoire diversity with age in humans [60, 61]. Thus, partly because of the increased total number of T cells in the human compared to mouse (3 × 10^11^ compared to 1–2 × 10^8^) [62–64], the CD8 T cell repertoire in human remains reasonably diverse and unlikely to develop holes [65], as we have shown in the mouse [7]. However, in very old individuals, clonal expansions and loss of the proliferative capacity of T cells result in more dramatic reduction of the human T cell repertoire [18, 63, 66, 67], making the results in aged mice relevant.

Another important question is whether VM are found in humans and whether they increase with age, as has been shown for the mouse. Importantly, although the phenotypic markers differ from VM in mouse, a population of CD8 T cells that functionally resembles VM has been identified in human, and these cells have been shown to increase with age in splenocytes and liver in human [39, 53]. For example, the data showed an increase in human VM cells in the spleen from ~ 5% at age 30 to ~ 20% at age 65 [53]. The studies reported here show VM cells play a major role in the response of aged mice to new infections, whereas the impact of human VM cells on responsiveness of the elderly to new infections is unknown. To add to the complexity of peripheral memory CD8 T cells in aging, a novel population of memory cells with a naïve phenotype has recently been shown to accumulate with age in humans [68]. It remains to be determined if a similar population is found in aged mice.

In our studies, the CD8 memory T cell population in aged SPF mice was dominated by VM cells. We hypothesized that aged mice that have experienced infections during their lifespan comparable to humans would have a higher ratio of TM to VM. It has been argued that VM are not an artifact of spf housing of mice because VM are generated and persist after bystander activation during deliberate, unrelated infections [56] However, a single infection in the mouse is not comparable to a lifetime of infections, including persistent infections, in human. Newly-developed “dirty” mouse models will allow testing the possibility that spf housing explains the large population of VM cells [69–72]. It has not yet been determined whether VM dominate the peripheral repertoire of CD8 T cells in antigen-experienced (dirty), aged mice.

The knowledge that T cell immunity is long-lived has raised the suggestion that aggressive immunization in youth and middle age would prevent the decline in repertoire and function of CD8 T cells associated with aging, and provide an approach to enhancing vaccination efficacy for the elderly. In support of this, it has been shown that priming of mice at an early age to influenza virus results in preservation of numbers, repertoire and function of virus-specific memory CD8 T cells [9, 73]. This is in stark contrast to the reduced repertoire diversity and impaired response of CD8 T cells that are observed following primary infection of aged mice [7, 9, 41]. Despite the apparent ability of VM cells to clear virus in the absence of specific immunization, it should be remembered that the response of TM out-competes the response of VM when directly compared [52, 55]. Thus, specifically increasing the population of TM by vaccination will likely reduce the impact of VM in aged, specific pathogen free mice to influenza virus infection, supporting the concept of vaccinations early in life. In light of the observation that VM differentiate into long-lived CM phenotype cells after priming [48], whereas TM develop preferentially into short-lived EM, it will be important in future studies to determine the contribution of VM and TM in response to infection in young mice and the contribution of each cell type to long-term maintenance of memory.

## Conclusions

In conclusion, the declining response of the elderly to new infections and vaccines is well-established and provides a compelling rationale for dissecting the impact of age-associated changes in the CD8 T cell repertoire and functional immune response to new infections, using the mouse model. Although the mouse model is robust, it is important to keep in mind differences between humans and mice and, where possible, validate findings in the mice with experiments in humans.

In the current studies, we have tested the hypothesis that in the face of declining numbers and repertoire diversity of naïve CD8 T cells in aged mice, responses are dominated by fortuitously cross-reactive memory cells. Adoptive transfer studies confirmed that memory CD8 T cells from influenza-naïve aged mice can respond to de novo infection with influenza virus in influenza-naïve aged mice with a highly constricted T cell receptor repertoire for the well characterized epitopes in C57BL/6 mice. Unexpectedly, the response was mediated by CD8 T cells with a virtual memory phenotype, rather than by true memory CD8 T cells previously generated in response to unrelated antigens. The VM CD8 T cells that dominated the repertoire in aged mice became functional granzyme B-producing effector cells and were able to clear influenza virus with a comparable kinetics to cells from young naïve mice. These data confirm the complexity of aging effects on the peripheral CD8 T cell repertoire.

## Methods

### Mice and viral infections

Female C57BL/6, B6.SJL-Ptprca Pepcb/BoyJ (B6.CD45.1), B6.PL-Thy1a/Cy (B6.CD90.1) and B6.129P2-Tcrb^tm1Mom^ Tcrd^tm1Mom^/J (TCRβδ^−/−^) were obtained from the Trudeau Institute animal facility or purchased from Jackson Laboratory and maintained under specific pathogen-free conditions. Often, but not always, impaired immune function has been attributed to the presence of large TCEs in the CD8 population [4, 7]. To avoid this complicating factor, peripheral blood lymphocytes of all aged mice were prescreened for major CD8 T cell Vβ expansions, and those that exhibited TCR Vβ8, Vβ7 or Vβ8.3 staining ± 4 SD over that observed with young C57BL/6 mice were omitted from the study. Mice were anesthetized with 2,2,2,-tribromoethanol and infected intranasally (i.n.) with 3000 EID_50_ A/HK-× 31 (× 31, H3N2). All experiments were approved by the Institutional Animal Care and Use Committee of the Trudeau Insitute.

### Lymphocyte isolation and flow cytometry

Lung tissue was prepared by coarsely chopping the tissue followed by incubation in a 0.5 mg/mL solution of collagenase D (Roche) and DNase (Sigma Aldrich) for 30–45 min at 37 °C. Lymphocytes were enriched from digested lung tissue by differential centrifugation, using a gradient of 40/80% Percoll (GE Healthcare). Single-cell suspensions were prepared from lymph nodes and spleens by dispersing the tissues through a 70 μm cell mesh. Single-cell suspensions were incubated with Fc-block (anti-CD16/32) for 15 min on ice followed by staining with MHC class I tetramer reagents for one hour at room temperature. Tetramer-labeled cells were then washed and stained with fluorochrome-conjugated antibodies against CD4, CD8α, CD19, CD90.2 (BD); CD44, CD45.1, CD45.2, CD62L (BioLegend); CD44 and CD49d (eBioscience) for 30 min on ice. MHC class I peptide tetramers specific for influenza virus NP_366_/D^b^, PA_224_/^Db^, PB1_703_/K^b^, PB1-F2_62_/D^b^, and NS2_114_/K^b^ were generated by the Trudeau Institute Molecular Biology Core Facility. For granzyme B staining, cells were fixed and permeabilized using the Cytofix/Cytoperm kit (BD) after tetramer and surface marker staining. Cells were stained with anti–granzyme B (or isotype control) antibody conjugated to PE (Invitrogen). Stained samples were run on a FACS Canto II or LSRII flow cytometer (BD Biosciences) and data were analyzed with FloJo software (TreeStar).

### Isolation of CD8 T cell subpopulations

Spleen and superficial lymph nodes were harvested from young (2–3 months) or aged (18–22 months) mice, processed into single-cell suspensions as described above, and further enriched by negative selection for CD8 cells using the BD Mouse CD8 T Lymphocyte Enrichment Kit. Sorting was performed on a BD Influx cell sorter with BD FACS Sortware software. Combinations of CD45 and CD90 alleles were chosen to allow discrimination of co-transferred populations, as shown in figures, where applicable.

In studies described in Figs. 1, 2 and 4, sorted cell populations were transferred, along with CD8-depleted splenocytes from young (2–3 month) B6.CD90.1 donors, into young TCR-deficient hosts (TCRβδ^−/−^) via intravenous injection. For the studies described in Fig. 1, CD8 T cells were enriched from pooled spleen and lymph nodes from individual naive aged (18–22 months) mice and sorted to isolate the CD44^High^ CD8 T cells. Total sorted cells, along with CD8 depleted splenocytes from young (2–3 month) B6.CD90.1 donors, were transferred into individual young TCRβδ^−/−^ hosts via intravenous injection. Recipient mice were infected i.n. with X-31 influenza virus (H3N2, 3000 EID_50_/mouse) 1 day after transfer. BAL, lung and spleen were harvested for analyses at day 12 following infection. Responding donor CD8 T cells were identified and enumerated through the use of antibody staining and MHC class I tetramers.

For studies described in Fig. 2, CD8 T cells were enriched from pooled spleen and lymph nodes from naïve aged (18–22 months) B6.CD45.1 or C57BL/6 mice and sorted to isolate the CD44^High^ and CD44^Low^ CD8 T cells. After sorting, the cells were transferred at either a 1:1 or 1:9 ratio of CD44^Low^:CD44^High^, along with CD8-depleted splenoctyes from young (2–3 month) B6.CD90.1 donors, into young TCRβδ^−/−^ hosts. Recipient mice were infected i.n. with X-31 influenza virus (H3N2, 3000 EID_50_/mouse) 1 day after transfer. BAL, lung and spleen were harvested for analyses at day 12 following infection. Responding donor CD8 T cells were identified and enumerated through the use of antibody staining and MHC class I tetramers.

For studies described in Fig. 4, CD8 T cells were enriched from pooled spleen and lymph nodes from naïve aged (18–22 months) B6.CD45.1 or B6.CD45.2 mice and sorted to isolate the TM (CD62L^High^CD44^High^CD49d^High^) and the VM (CD62L^High^CD44^High^CD49d^Low^) CD8 T cells. The sorted cells were adoptively transferred into young TCRβδ^−/−^hosts at a 1:1 ratio. Recipient mice were infected i.n. with X-31 influenza virus (H3N2, 3000 EID_50_/mouse) 1 day after transfer. BAL, lung and spleen were harvested for analyses at day 12 following infection. Responding donor CD8 T cells were identified and enumerated through the use of antibody staining and MHC class I tetramers.

For studies described in Fig. 5, CD8 T cells were enriched from pooled spleen and lymph nodes from naïve young (2–3 months) or naïve aged (18–22 months) mice and sorted to isolate the CD44^Low^ CD8 T cells or the CD62L^High^CD44^High^CD49d^Low^ CD8 T cells, respectively. Sorted cells from young or aged mice were transferred into young TCRβδ^−/−^ hosts. Recipient mice were infected i.n. with X-31 influenza virus (H3N2, 3000 EID_50_/mouse) 1 day after transfer. BAL, lung and spleen were harvested for analyses at days 6 and 9 following infection. Responding donor CD8 T cells were identified and enumerated through the use of antibody staining and MHC class I tetramers, and evaluated for Granzyme B expression.

For studies described in Fig. 6, spleen and superficial lymph nodes were harvested from naïve young (2–3 months) or aged (18–22 months) mice, pooled, and enriched by negative selection for CD8^+^ cells using the BD Mouse CD8 T Lymphocyte Enrichment Kit. 2.5 × 10^6^ enriched CD8 T cells from either young or aged mice were transferred into young TCRβδ^−/−^ hosts. Recipient mice were infected i.n. with X-31 influenza virus (H3N2, 3000 EID_50_/mouse) 1 day after transfer. Lungs were harvested from recipient mice at days 8 and 10 and processed for viral titers. In a separate control experiment, young TCRβδ^−/−^ with no T cell transfer and young C57BL/6 mice were infected i.n. with X-31 influenza virus (H3N2, 3000 EID_50_/mouse). Lungs were harvested at days 8 and 10 and processed for viral titers.

### Measurement of viral load

Whole lung tissue was harvested in PBS at the indicated times, homogenized, and stored at − 70 °C. Virus titers were measured with a standard plaque assay by infecting MDCK cell monolayers with serial 5-fold dilutions of lung suspension in duplicate. Eighteen to twenty-four hours after infection, monolayers were washed and fixed with 80% acetone in water. Infected cell clusters were detected with a biotin-labeled mouse anti-influenza A monoclonal antibody (Chemicon), followed by staining with streptavidin-AP, and visualized with Sigma Fast BCIP/NBT substrate (Sigma). The number of viral-foci units (VFUs) was counted, and the data were shown as the VFU/lung.

### Statistical analysis

Statistical analysis was performed with GraphPad Prism 5 software (GraphPad, San Diego, CA). Differences were considered significant at *p* value < 0.05.

## Additional file

Additional file 1: **Table S1.** Cell transfer number and tetramer frequency for individual aged mice transfer study. (DOCX 14 kb)

## Abbreviations

BAL: Bronchoalveolar lavage
CM: Central memory
EM: Effector memory
NP: Nucleoprotein
TM: “True” memory
T_RM_: Resident memory
VM: Virtual memory
