# Correction to: Virtual memory cells make a major contribution to the response of aged influenza-naïve mice to influenza virus infection

**DOI:** 10.1186/s12979-018-0123-x

**Published:** 2018-08-21

**Authors:** Kathleen G. Lanzer, Tres Cookenham, William W. Reiley, Marcia A. Blackman

**Affiliations:** Trudeau Institute, 154 Algonquin Avenue, Saranac Lake, NY 12983 USA

## Correction

In the original publication of this article [1] there is an error in Fig. 3a (Fig. 1 here) as the labels “TM” and “VM” are in the wrong order for the last plot. The CD49d high cells should be “TM” and the CD49d low cells should be “VM”.

**Fig. 1:**
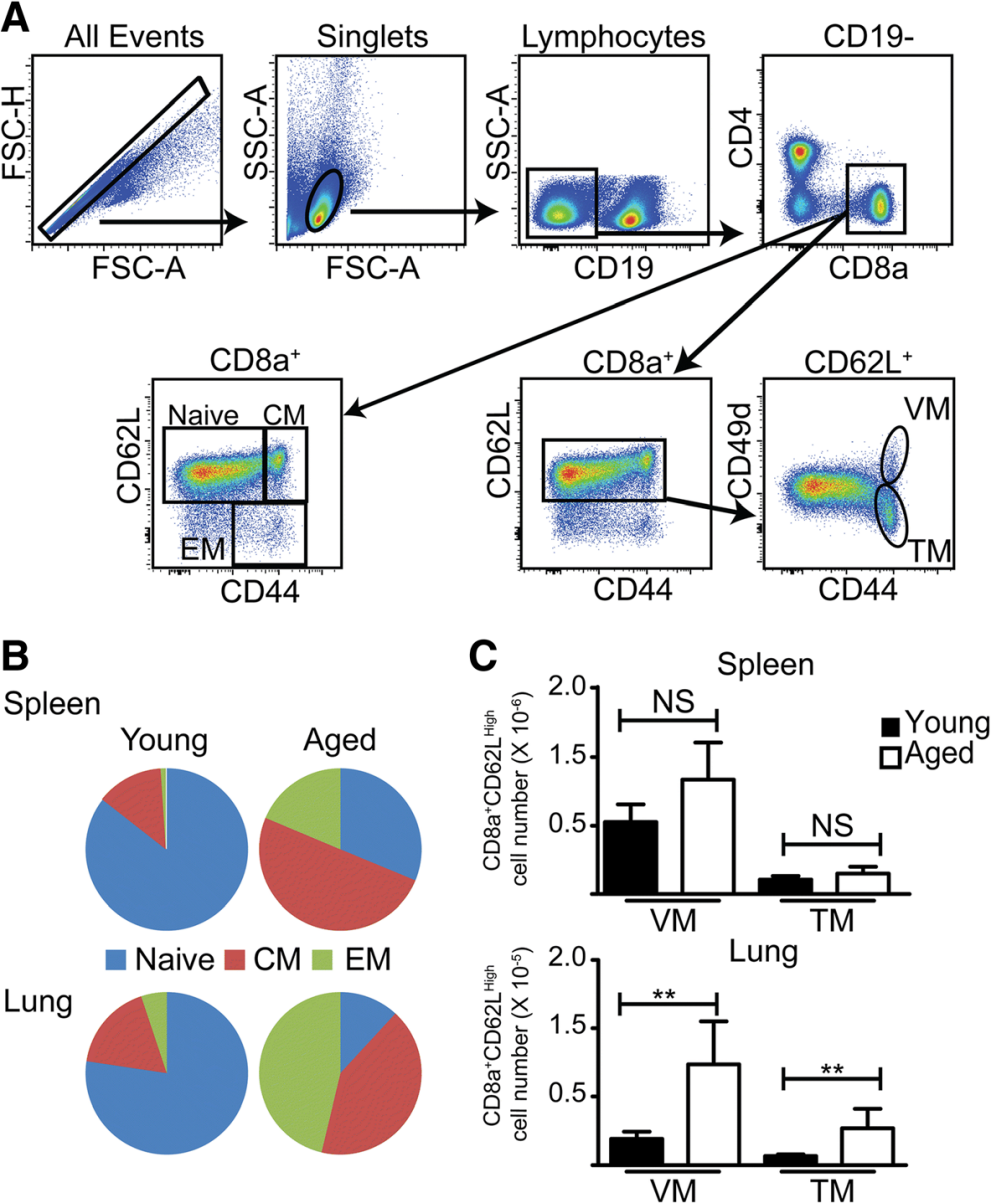
Incorrect version of Figure 3 as published on 8 August 2018

In this correction article the incorrect and correct figure (Fig. 2 here) are published.

**Fig. 2:**
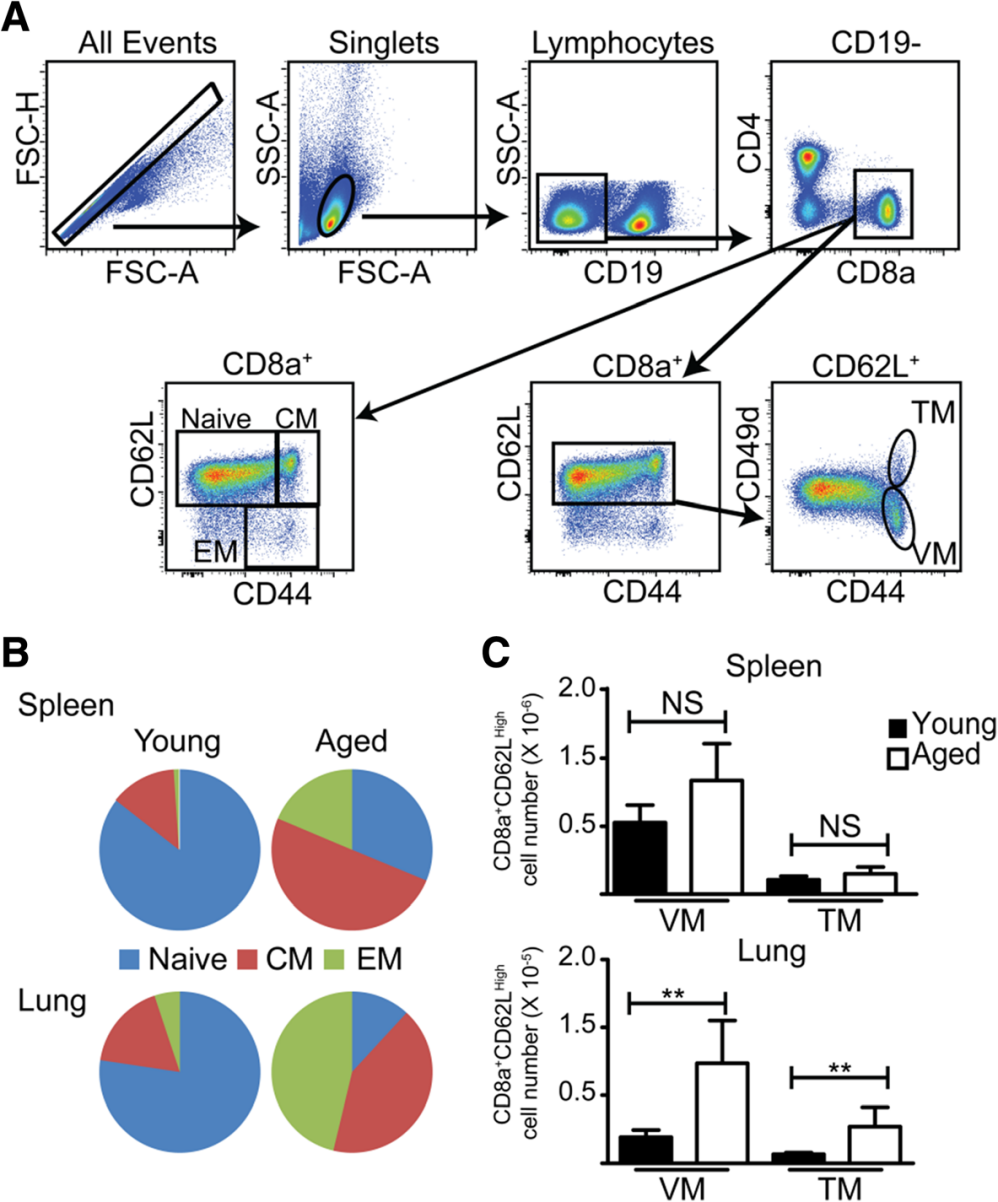
Corrected version of Figure 3
