# Supplementary material for: Virtual memory cells make a major contribution to the response of aged influenza-naïve mice to influenza virus infection
Source: Immun Ageing. 2018 Aug 8;15:17. doi: 10.1186/s12979-018-0122-y (PMC6081820; doi:10.1186/s12979-018-0122-y)
Supplement: Supplementary file 1 — Table S1. Cell transfer number and tetramer frequency for individual aged mice transfer study. (DOCX 14 kb) [file 12979_2018_122_MOESM1_ESM.docx]

**Table 1**

Cell transfer number and tetramer frequency for individual aged mice transfer study

|  |  | Tetramer Frequency* | | | | |
| --- | --- | --- | --- | --- | --- | --- |
| Mouse | Sort Count | NP_366_/D^b^ | PA_2246_/D^b^ | PB1_703_/K^b^ | PB1-F2_62_/D^b^ | NS2_114_/K^b^ |
| 1 | 6.50 x 10^5^ |  |  | 21.6 |  |  |
| 2 | 1.30 x 10^6^ |  | 7.03 | 29.7 |  | 0.98 |
| 3 | 8.90 x 10^5^ |  |  | 1.46 |  |  |
| 4 | 1.10 x 10^6^ |  |  | 19.4 |  |  |
| 5 | 2.50 x 10^5^ |  |  | 10.2 |  |  |
| 6 | 6.50 x 10^6^ |  |  | 5.27 |  |  |
| 7 | 1.80 x 10^6^ |  |  |  |  |  |
| 8 | 9.10 x 10^5^ | 0.99 | 13.9 | 3.3 |  |  |
| 9 | 5.50 x 10^5^ | 31.6 |  | 7.62 | 0.4 |  |
| 10 | 1.10 x 10^6^ |  | 1.5 | 37 |  |  |
| 11 | 1.05 x 10^6^ |  | 22 | 2.9 |  |  |
| 12 | 1.80 x 10^6^ |  |  | 7.6 |  | 0.6 |
| 13 | 1.20 x 10^6^ |  |  |  |  |  |
| 14 | 4.00 x 10^5^ |  | 22 | 0.7 |  |  |
| 15 | 7.50 x 10^5^ |  |  | 34 |  |  |
| 16 | 6.50 x 10^5^ |  | 0.7 | 7.8 |  |  |
| 17 | 1.70 x 10^6^ |  |  | 2.2 |  |  |
| 18 | 5.80 x 10^5^ | 0.6 |  |  |  |  |
| 19 | 1.10 x 10^6^ |  | 1.3 | 15 |  |  |

*Frequency of tetramer-specific donor CD8 T cells at day 12 post-infection
